# Identifying the Hub Genes of Glioma Peritumoral Brain Edema Using Bioinformatical Methods

**DOI:** 10.3390/brainsci12060805

**Published:** 2022-06-19

**Authors:** Yuxi Wu, Zesheng Peng, Haofei Wang, Wei Xiang

**Affiliations:** Department of Neurosurgery, Union Hospital, Tongji Medical College, Huazhong University of Science and Technology, Wuhan 430022, China; m202075979@hust.edu.cn (Y.W.); d202081762@hust.edu.cn (Z.P.); m201875614@hust.edu.cn (H.W.)

**Keywords:** glioma, peritumoral edema, inflammatory, gene, data integration analysis

## Abstract

Glioma peritumoral brain edema (GPTBE) is a frequent complication in patients with glioma. The severity of peritumoral edema endangers patients’ life and prognosis. However, there are still questions concerning the process of GPTBE formation and evolution. In this study, the patients were split into two groups based on edema scoring findings in the cancer imaging archive (TCIA) comprising 186 TCGA-LGG patients. Using mRNA sequencing data, differential gene (DEG) expression analysis was performed, comparing the two groups to find the key genes affecting GPTBE. A functional enrichment analysis of differentially expressed genes was performed. Then, a protein–protein interaction (PPI) network was established, and important genes were screened. Gene set variation analysis (GSVA) scores were calculated for major gene sets and comparatively correlated with immune cell infiltration. Overall survival (OS) was analyzed using the Kaplan–Meier curve. A total of 59 DEGs were found, with 10 of them appearing as important genes. DEGs were shown to be closely linked to inflammatory reactions. According to the network score, IL10 was in the middle of the network. The presence of the IL10 protein in glioma tissues was verified using the human protein atlas (HPA). Furthermore, the gene sets’ GSVA scores were favorably linked with immune infiltration, particularly, with macrophages. The high-edema group had higher GSVA scores than the low-edema group. Finally, Kaplan–Meier analysis revealed no differences in OS between the two groups, and eight genes were found to be related to prognosis, whereas two genes were not. GPTBE is linked to the expression of inflammatory genes.

## 1. Introduction

Gliomas are the most frequent primary central nervous system tumors, accounting for around half of all intracranial tumors [1]. Even after standard treatment, such as maximum surgical resection, radiotherapy, and chemotherapy, glioma patients’ prognosis varies greatly, and the median overall survival of glioblastoma (GBM) patients is only 19 months [2,3]. Glioma peritumoral brain edema (GPTBE) is a common symptom in these patients and might impair a patient’s prognosis [4,5]. In individuals with significant peritumoral edema, increased intracranial pressure might ensue, potentially leading to a life-threatening herniation [6]. As a result, GPTBE diagnosis and therapy are critical.

A swelling caused by an improper distribution of water in the brain parenchyma is known as brain edema. Numerous studies have been conducted to investigate the molecular pathways causing GPTBE development in glioma patients. Early research pointed to vasogenic edema as the primary cause, which is mostly linked to a breakdown of the blood–brain barrier (BBB) [7]. In a growing number of studies, GPTBE has been linked to the invasive potential of gliomas, and it is thought that the invasion of tumor cells leads to the remodeling of surrounding tissues. Brain edema is caused by the proteins aquaporin 4 (AQP4), metalloproteinase 9 (MMP9), and vascular endothelial growth factor (VEGF). VEGF expression has been shown to promote tumor neovascularization while decreasing vascular permeability [8]. MMP9 destroys the extracellular matrix thus providing enough room for AQP4, an essential channel involved in cell water transport [9,10,11]. The basic processes of GPTBE remain unknown, as do the exact molecular pathways.

The cancer imaging archive (TCIA) is a master site for cancer imaging analysis [12]. The TCIA has magnetic resonance (MR) images that have been linked to cancer genome atlas (TCGA) partial samples and to findings of professional radiologists’ analyses. This offered us a handy way to research GPTBE. The goal of this study was to look at the TCGA-LGG mRNA sequencing data as well as to edema score findings of brain MR in related patients to see whether there were any genes that affected GPTBE.

## 2. Materials and Methods

### 2.1. Data Sources

TCIA provided data for cancer imaging assessments, which included TCGA-LGG (https://wiki.cancerimagingarchive.net/pages/viewpage.action?pageId=24282890, accessed on 2 March 2022) [6]. Visually accessible Rembrandt images (VASARI) were employed for radiographic evaluation of parameters, which were all assessed by experienced radiologists (Supplementary file S1). For analysis, data in raw “counts” format were collected from the TCGA website.

### 2.2. Assessments of Image Features

The “F14” feature of VASARI is the proportion of edema, which includes 10 options (0 = -, 1 = n/a, 2 = None (0%), 3 = <5%, 4 = 6–33%, 5 = 34–67%, 6 = 68–95%, 7 = >95%, 8 = All (100%), 9 = Indeterminate). Supplementary file S2 reports a detailed discussion of this feature. Before data analysis, samples with options 0, 1, and 9 were deleted. Samples with options 2 or 3 were placed in the low-edema group, whereas samples with options 4, 5, 6, 7, or 8 were placed in the high-edema group.

### 2.3. Differentially Expressed Gene (DEGs) Analysis

We used the R program “DESeq2” to extract differential information and evaluate the significance of each gene difference in various groups. Gene names were searched on the Genecards website, and protein-coding genes were kept as DEGs, while other genes were left out. A *p*-value < 0.05 and a log2|fold change| > 1 were deemed significant.

### 2.4. Gene Ontology (GO) and Kyoto Encyclopedia of Genes and Genomes (KEGG) Analysis

GO and KEGG analyses were performed to functionally annotate DEGs by the “clusterprofiler” package of R software and study the probable roles of differentially expressed genes. Cellular components (CC), biological processes (BP), and molecular function (MF) were included in the GO study. Entries with a *p*-value < 0.05 and an adjusted *p* value < 0.1 were considered.

### 2.5. Correlation Analysis and PPI Network Construction

Spearman correlation analysis was utilized to examine the connection between each expressed gene, in conjunction with a quantitative analysis of differential gene expression. STRING (https://string-db.org/, accessed on 5 March 2022). We also studied the biological interactions between proteins with varied gene expression patterns. Using the “cytohubba” plugin in Cytoscape, hub genes in the protein–protein interaction (PPI) network were discovered using the degree technique. Central node components can be thought of as core proteins and essential hub genes with vital physiological roles.

### 2.6. Survival Analysis of Hub Genes

Patients in the TCGA-LGG cohort were separated into two groups, high expressors and low expressors, based on the median expression levels of important genes, and survival curves were produced based on overall survival (OS). With time information for each sample, a Kaplan–Meier (KM) survival analysis was conducted using the R package “survival.” Statistical significance was determined using the log-rank test. The human protein atlas (HPA) [13] provided information on protein expression of key genes measured by immunohistochemistry.

### 2.7. Immune infiltration analysis

Immune impact is directly linked to inflammatory reactions. The gene set variation analysis (GSVA) score indicates the integrated level of genomic expression, which is inversely proportional to genome expression. As a result, a higher GSVA score in a tumor group might suggest a greater overall expression of that gene set. The R package “GSVA” was used to construct GSVA scores in order to look into the relationship between central gene sets and immune cell infiltration. The Gene Set Caner Analysis (GSCA) website (http://bioinfo.life.hust.edu.cn/GSCA, accessed on 7 March 2022) reports the analysis and visualization of the infiltration of 24 immune cells [14]. The correlation coefficient was used to reflect the relationship between immune cell infiltrates and gene set expression levels, which was analyzed using Spearman correlation analysis. The false discovery rate (FDR) was used to alter the *p*-value [15].

### 2.8. Statistical Analysis

Excel was used to process the raw data. The SPSS 23.0 program was mostly used to analyze clinical data. The chi-square test was used to compare categorical data. The Student’s t test or the Kruskal–Wallis test was used to calculate statistical significance for continuous variables between two groups or more than two groups. R was used to sequence the data, analyze them, and visualize them (v3.6.1). The correlation between each differential gene was determined using the Spearman correlation approach; *p* < 0.05 was regarded as statistically significant.

## 3. Results

### 3.1. Clinical Features

**Figure 1:**
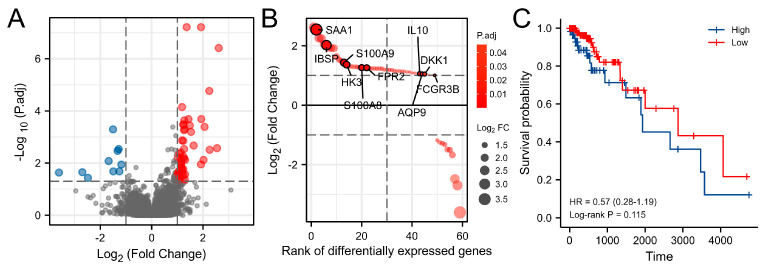
Differences in survival time and gene expression between the two groups. (**A**) Differentially expressed genes (DEGs). Genes with increasing expression were represented in red, whereas genes with decreased expression were represented in blue. (**B**) Ranking plot of DEGs. (**C**) Kaplan–Meier curves for the two groups.

In total, 178 patients with imaging data in TCGA-LGG were sorted into two groups according to our grouping technique. The low-edema group included 116 patients, while the high-edema group comprised 62 individuals. As reported in Table 1, gender, tumor site, pathological grade, lactate dehydrogenase 1 (IDH1) status, 1p/19q status, and P53 status did not change between the two groups of patients, as reported in Table 1, with statistical differences only in age (*p* = 0.022). The two groups of patients had the same OS rate (Figure 1C, *p* = 0.115).

### 3.2. Differentially Expressed Genes (DEGs) Analysis

The raw mRNA expression data were processed using the R software to acquire the genes differentially expressed in the two groups in order to understand genomic expression differences. Figure 1A,B revealed a total of 59 genes differentially expressed genes at a significant level. Supplementary file S3 shows the results of the original analytical computations.

### 3.3. Functional Analysis of DEGs

**Figure 2:**
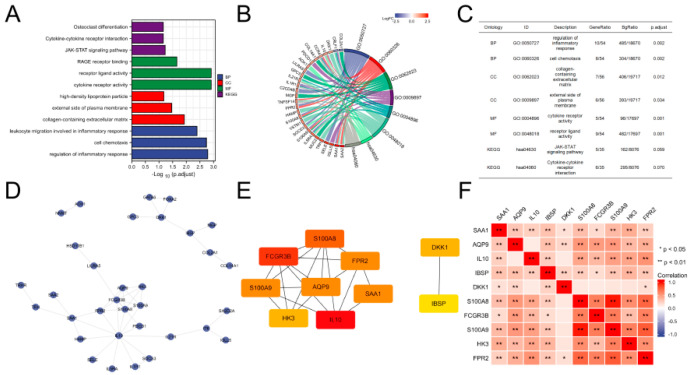
Functional enrichment analysis of differentially expressed genes. (**A**–**C**) GO and KEGG analysis. (**D**,**E**) Protein interaction network diagram. The darker the template color, the greater the interaction score. (**F**) Heatmap of key genes with expression correlations.

GO and KEGG analyses are useful tools for determining the likely function of genes. Figure 2 depicts the changes in genes related to BP, MF, CC according to KEGG. The original functional enrichment analysis data are reported in Supplementary file S4. Inflammatory response, cell chemotaxis, and leukocyte movement were all controlled by BP. MF was related to cytokine receptor activity, receptor ligand activity, and receptor binding of advanced glycation end-products (RAGE). Collagen-containing extracellular matrix, plasma membrane’s exterior side, and high-density lipoprotein particles were found in relation to CC. The Janus kinase signal transducer and activator of transcription (JAK–STAT) signaling pathway, cytokine–cytokine receptor interactions, and osteoclast differentiation were all heavily represented in the inflammation-related activities of KEGG.

### 3.4. PPI Network Construction and Key Gene Identification

**Figure 3:**
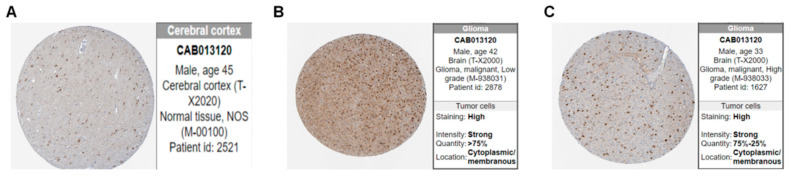
Immunohistochemical image of IL10 on the HPA website. (**A**) Normal tissue. (**B**) Low-grade glioma. (**C**) High-grade glioma.

Proteins are essential for carrying out biological tasks. A hypothetical PPI network was built using the STRING website, as illustrated in Figure 2D. Figure 2E shows that 10 important genes were ruled out (IL10, FCGR3B, S100A8, AQP9, S100A9, FPR2, SAA1, HK3, DKK1, and IBSP). Table 2 shows the detailed results for each of the 10 genes. The most important gene was interleukin 10 (IL10); immunohistochemistry of its protein is displayed in Figure 3. As indicated in Figure 2F, Dickkopf Wnt signaling pathway inhibitor 1 (DKK1) and integrin-binding sialoprotein (IBSP) were substantially less linked with the expression of other molecules.

### 3.5. Key Genes Prognostic Analysis

**Figure 4:**
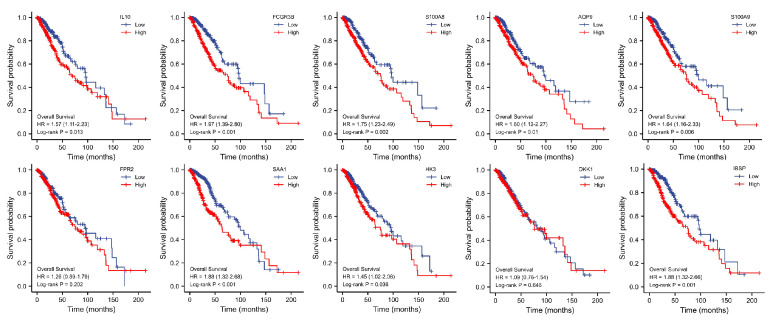
Survival analysis in relation to 10 key genes.

Eight of the ten key genes were linked to poor prognosis in LGG patients, including IL10 (*p* = 0.013), S100A8 (*p* = 0.002), AQP9 (*p* = 0.01), Fc gamma receptor IIIb (FCGR3B) (*p* < 0.001), S100A9 (*p* = 0.006), serum amyloid A1 (SAA1) (*p* < 0.001), hexokinase 3 (HK3) (*p* = 0.036), DKK1 (*p* = 0.646), IBSP (*p* = 0.001), and formyl peptide receptor 2 (FPR2) (*p* = 0.202), as shown in Figure 4.

### 3.6. Immune Infiltration Analysis of Gene Sets

**Figure 5:**
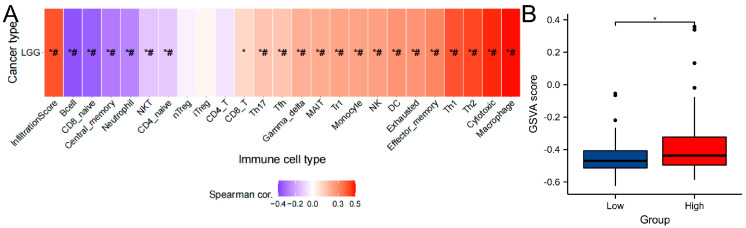
Immune cell infiltration analysis. (**A**) Correlation of immune infiltration based on the GSVA score. (**B**) GSVA scores were compared between groups. *: *p* value < 0.05; #: FDR < 0.05.

Infiltration of the immune system and the inflammatory response are linked. By using the GSCA online tool, we found that macrophages were the cells most strongly correlated with gene sets, while B cells were the least correlated. The infiltration scores revealed that gene sets overall expression was positively correlated with the degree of immune infiltration, based on the GSVA scores of key gene sets (Figure 5A, Supplementary file S5). The GSVA scores of gene sets were greater in the high-edema group (*p* < 0.05, Figure 5B) than in the low-edema group.

## 4. Discussion

GPTBE causes the fluid content of peritumoral tissue to rise. Gliomas account for approximately 80% of intracranial malignancies, and GPTBE is a prevalent occurrence [1,2,3]. Most studies concur that the presence of GPTBE in gliomas is linked to tumor aggressiveness and patient prognosis, and that severe GPTBE can be fatal [4,16]. GPTBE has a wide range of effects on glioma patients, with some showing severe edema, and others showing little or no edema. As a result, studying the underlying molecular pathways aids in gaining a better understanding of GPTBE.

By merging the brain edema scores of glioma patients with high-throughput sequencing data of related patients, this study looked at possible chemicals that influence GPTBE. Our findings show that inflammation-related chemicals, as well as the inflammatory response that results from them, are implicated in the development and progression of GPTBE. Hormone therapy is a therapeutic option for GPTBE [17]. This observation is significant because it adds to the growing body of information supporting the role of immunological inflammation in GPTBE [4,5,6,18]. Furthermore, some studies suggest that GPTBE influences glioma patients’ prognosis. However, our research found that the degree of GPTBE was not linked to glioma patients’ survival. We believe the variance is due to the varying GPTBE assessment standards utilized by different research studies as well as the different sample sizes. As a result, the mechanism of GPTBE remains a point of contention [4].

Currently, GPTBE is thought to cause mainly vasogenic and cellular edema [19]. The natural BBB is disrupted by the brain tumor tissue, which leads to increased capillary permeability and hence water buildup [20]. Increased VEGF expression has been established as a significant factor in GPTBE. VEGF is aberrantly elevated in GBM, causing pathological disruption of the BBB, further allowing leakage of neurotoxic molecules, interfering with tumor microenvironment homeostasis, and contributing to poor patient outcome [21,22,23]. Extensive microvascular development is induced by VEGF acting on appropriate receptors on vascular endothelial cells, and immature vascular structure results in fluid exudation [7,22]. According to recent studies [6,24], the STAT–VEGF pathway is critical for GPTBE. Our differentially expressed genes were also enriched in STAT-related pathways, indicating that the STAT–VEGF pathway plays a role. Inflammatory reactions can also compromise the BBB, resulting in cerebral hematomas in various neurological illnesses [7,25], and the role of immune cells in the tumor microenvironment is critical. The fast growth and invasion of glioma cells establishes a hypoxic–ischemic environment in the tumor’s local area, and the accumulating toxic metabolites cause energy metabolism problems in the peritumoral normal cells, resulting in cytotoxic edema [25]. In malignancies, the connection between abnormal metabolic pathways and immune cell infiltration is complicated and varied. In the glioma microenvironment, tumor-associated macrophages/microglia (TAMs) are the most abundant stromal cells [25,26]. TAMs enhance angiogenesis by generating a variety of pro-angiogenic and chemokine factors [26]. The degree of TAM infiltration was shown to be positively linked with the degree of PTBE penetration in this investigation. The infiltration of cytotoxic T lymphocytes (CTLs) and macrophages was larger in the high-edema group than in the low-edema group, according to our findings. Increased CTLs with macrophages will release a plethora of cytokines to destroy tumor cells, resulting in an increase in metabolites and chemotaxis [25]. This might lead to the establishment of peritumoral edema. Two forms of glioma TAMs have been identified: anti-inflammatory M2-type macrophages and activated pro-inflammatory M1-type macrophages. TAMs, under the influence of glioma cells, appear to play a major role in tumor angiogenesis, local development, and invasion, according to an increasing number of data [25,26,27]. Thus, we believe that peritumoral edema is the result of multifactorial participation and multidimensional control, with immunological imbalance being a key connection.

The 10 major genes that were elevated have diverse functions in the immune inflammatory response balance. IL-10 is a potent anti-inflammatory cytokine with a wide range of functions in the immune system and inflammation. In the setting of cancer, IL10 causes immunosuppression, which decreases T cell proliferation by inhibiting antigen-presenting cells. IL10 binds to its corresponding receptor, causing STAT3 phosphorylation through JAK1 and STAT2, which stimulates the proliferation of glioma cells [24,28]. Activated STAT3 in glioma cells can also alter angiogenesis and GPTBE through the regulation of VEGF. Calprotectin (S100A8/A9) is generated from neutrophil and macrophage calcium-binding proteins [18,29]. It exerts proinflammatory, antibacterial, oxidant scavenging, and apoptosis-inducing properties. The SAA1 gene codes for a key acute-phase protein that is highly produced in response to tissue injury and inflammation. Through autocrine and paracrine actions, SAA proteins can promote tumor development. SAA not only influence tumor cell migration, invasion, angiogenesis, and IL-8 release in T98G and A172 cell lines, but also increase cell proliferation and the formation of nitric oxide and reactive oxygen species [30]. FPR2 is a strong chemoattractant for neutrophils. By decreasing the levels of VEGF, Liu et al. showed that silencing the FPR2 gene decreased U87 cell proliferation, migration, and invasion [31]. FCGR3B is a low-affinity receptor for the Fc region of immunoglobulin (IgG) that can be used to collect immune complexes in the bloodstream. HK3 is a key regulating enzyme in the first stage of the glucose metabolic pathway. Hexokinase is required for tumor cell decryption during aerobic glycolysis. AQPs are membrane channel proteins that regulate water and solute transport across the phospholipid bilayer. AQP4 is expressed in the end-feet of astrocytes, regulates osmolarity, and has a polarized pattern [9,32,33]. In pathological situations, the distribution and expression of AQP4 are aberrant, resulting in cerebral edema. Mou et al. discovered that aberrant APQ4 expression was frequently accompanied by alterations in the expression of other molecules, such as VEGF overexpression [9]. AQP4 is a critical molecule in GPTBE research, and we discovered that highly expressed AQP9 was also linked to GPTBE in our study. AQP9 is a protein that is expressed in astrocytes and is involved in brain energy metabolism [34]. In a rat intracerebral hemorrhage model, downregulation of AQP9 was also found to limit angiogenesis [35]. The above molecular clusters may have an indirect impact on PTBE through tumor angiogenesis and immunological modulation. The Wnt signaling pathway is inhibited by DKK1 [36]. In a number of studies [23,26], Wnt signaling has been linked to GBM development, invasion, and treatment resistance. IBSP is a focal adhesion protein that aids in cell adhesion and migration on the cell surface [37]. In gliomas, the clinical importance of IBSP is unclear. According to studies, IBSP can form a trimolecular complex with integrins and MMP2, speeding up local matrix breakdown and cancer cell invasion [11]. As a result, from an indirect consideration of molecular function, DKK1 and IBSP may be involved in degrading the extracellular matrix, thereby providing space for GPTBE [37,38]. All these differentially expressed genes were identified in previous GPTBE research, demonstrating that GPTBE is caused by multifactorial, multidimensional changes in the tumor microenvironment.

Despite the novel nature of our research methods, there are numerous limitations to our study. First, GBM samples were left out since TCIA lacks GBM data, which might have resulted in a bias [5]. Second, these genes were not tested in glioma patients with large edemas. Finally, the expression of these genes varies by tumor tissue, and there are no direct investigations into peritumoral edematous tissues [39,40]. Finally, we used a combination of imaging and sequencing data to examine putative GPTBE compounds, utilizing multi-dimensional data in a novel way. Our findings suggest that immunological inflammatory responses are involved in the production of GPTBE.

## 5. Conclusions

Our findings showed that changes in the glioma immune microenvironment are highly linked to the extension of GPTBE, based on an integrated study of TCIA and TCGA data. IL10 may play a significant role in the pathogenesis of GPTBE by altering the local immunological status. We discovered that the degree of GPTBE was unrelated to OS in the studied research population. The complex molecular mechanisms underlying GPTBE need to be investigated further.

## Figures and Tables

**Table 1:** Clinical characteristics of the two groups.

Characteristic	Low Edema Group	High Edema Group	*p*
n	116	62	
Gender, n (%)			0.354
Female	50 (28.7%)	33 (19%)	
Male	62 (35.6%)	29 (16.7%)	
Laterality, n (%)			0.787
Left	55 (32%)	27 (15.7%)	
Midline	2 (1.2%)	1 (0.6%)	
Right	54 (31.4%)	33 (19.2%)	
Tumor location, n (%)			0.721
Posterior Fossa, Cerebellum	1 (0.6%)	0 (0%)	
Supratentorial, Frontal Lobe	64 (36.8%)	38 (21.8%)	
Supratentorial, Not Otherwise	3 (1.7%)	1 (0.6%)	
Supratentorial, Occipital Lobe	0 (0%)	1 (0.6%)	
Supratentorial, Parietal Lobe	13 (7.5%)	6 (3.4%)	
Supratentorial, Temporal Lobe	31 (17.8%)	16 (9.2%)	
Histologic grade, n (%)			0.094
G2	65 (37.4%)	27 (15.5%)	
G3	47 (27%)	35 (20.1%)	
IDH1, n (%)			1.000
Wild	28 (15.8%)	15 (8.5%)	
Mutant	88 (49.7%)	46 (26%)	
1p/19q co-del status, n (%)			0.377
Wild	85 (48.6%)	42 (24%)	
Mutant	28 (16%)	20 (11.4%)	
TP53, n (%)			0.870
Mutant	60 (33.9%)	30 (16.9%)	
Wild	56 (31.6%)	31 (17.5%)	
Age, median (IQR)	39 (30, 52.25)	48.5 (37, 57)	0.022

IDH, isocitrate dehydrogenase, IQR, interquartile range.

**Table 2:** Top 10 genes in the network ranked by the Degree method.

Rank	Name	Score
1	IL10	12
2	FCGR3B	8
3	S100A8	6
4	AQP9	5
4	S100A9	5
4	FPR2	5
4	SAA1	5
8	HK3	4
8	DKK1	4
10	IBSP	3

## Data Availability

The data sets supporting the conclusions of this article are available in the public databases The Cancer Genome Atlas (TCGA, https://portal.gdc.cancer.gov/, accessed on 5 February 2022), the Cancer Imaging Archive (TCIA, https://www.cancerimagingarchive.net, accessed on 1 March 2022), and the Gene Set Caner Analysis (GSCA, http://bioinfo.life.hust.edu.cn/GSCA, accessed on 7 March 2022).

## Supplementary Materials

The following supporting information can be downloaded at: https://www.mdpi.com/article/10.3390/brainsci12060805/s1. Supplementary file S1: Evaluation outcomes of TCGA-LGG patients in TCIA. Supplementary file S2: VASARI MR feature. Supplementary file S3: Differentially expressed genes. Supplementary file S4: Results of gene enrichment analysis. Supplementary file S5: Results of GSCA correlation statistics.

## Abbreviations

AQPs	Aquaporins
BBB	Blood–brain barrier
BP	Biological processes
CC	Cellular components
CTLs	Cytotoxic T cells
DEGs	Differential genes
DKK1	Dickkopf Wnt signaling pathway inhibitor 1
FCGR3B	Fc gamma receptor IIIb
FDR	False discovery rate
FPR2	Formyl peptide receptor 2
GBM	Glioblastoma
GPTBE	Glioma peritumoral brain edema
GSCA	Gene Set Caner Analysis
GSVA	Gene set variation analysis
GO	Gene Ontology
HK3	Hexokinase 3
HPA	Human protein atlas
IBSP	Integrin binding sialoprotein
IDH1	Lactate dehydrogenase 1
IL10	Interleukin 10
JAK	Janus kinase
KEGG	Kyoto Encyclopedia of Genes and Genomes
KM	Kaplan–Meier
LGG	Low-grade gliomas
MF	Molecular function
MMP	Metalloproteinase
MR	Magnetic resonance
OS	Overall survival
PPI	Protein–protein interaction
PTBE	Peritumoral brain edema
RAGE	Receptor of advanced glycation end-products
SAA1	Serum amyloid A1
STAT	Signal transducer and activator of transcription
TAMs	Tumor associated macrophages
TCGA	The cancer genome atlas
TCIA	The Cancer Imaging Archive
VASARI	Visually accessible Rembrandt images
VEGF	Vascular endothelial growth factor
