# Supplementary material for: Identifying the Hub Genes of Glioma Peritumoral Brain Edema Using Bioinformatical Methods
Source: Brain Sci. 2022 Jun 19;12(6):805. doi: 10.3390/brainsci12060805 (PMC9221376; doi:10.3390/brainsci12060805)
Supplement: Supplementary file 1 [file brainsci-12-00805-s001.zip › Supplementary file S1.pdf]

	f14
TCGA-CS-4938	2
TCGA-CS-4941	6
TCGA-CS-4942	2
TCGA-CS-4943	3
TCGA-CS-4944	3
TCGA-CS-5390	3
TCGA-CS-5393	3
TCGA-CS-5394	4
TCGA-CS-5395	2
TCGA-CS-5396	5
TCGA-CS-5397	3
TCGA-CS-6186	5
TCGA-CS-6188	2
TCGA-CS-6290	3
TCGA-CS-6665	4
TCGA-CS-6666	2
TCGA-CS-6667	2
TCGA-CS-6668	2
TCGA-CS-6669	6
TCGA-DU-5849	3
TCGA-DU-5851	4
TCGA-DU-5852	4
TCGA-DU-5853	2
TCGA-DU-5854	5
TCGA-DU-5855	3
TCGA-DU-5871	4
TCGA-DU-5872	3
TCGA-DU-5874	2
TCGA-DU-6395	6
TCGA-DU-6397	3
TCGA-DU-6399	2
TCGA-DU-6400	4
TCGA-DU-6401	4
TCGA-DU-6402	1
TCGA-DU-6404	3
TCGA-DU-6405	2
TCGA-DU-6407	3
TCGA-DU-6408	5
TCGA-DU-6410	3
TCGA-DU-6542	5
TCGA-DU-7008	5
TCGA-DU-7010	7
TCGA-DU-7013	2
TCGA-DU-7014	5
TCGA-DU-7015	3
TCGA-DU-7018	4
TCGA-DU-7019	4
TCGA-DU-7294	5
TCGA-DU-7298	4
TCGA-DU-7299	2

TCGA-DU-7300	5
TCGA-DU-7301	2
TCGA-DU-7302	5
TCGA-DU-7304	3
TCGA-DU-7306	3
TCGA-DU-7309	5
TCGA-HT-7684	3
TCGA-HT-7681	n/a
TCGA-HT-7686	3
TCGA-HT-7687	2
TCGA-HT-7688	2
TCGA-HT-7689	6
TCGA-HT-7690	3
TCGA-HT-7691	2
TCGA-HT-7692	7
TCGA-HT-7693	2
TCGA-HT-7694	6
TCGA-HT-7695	4
TCGA-HT-7854	2
TCGA-HT-7855	3
TCGA-HT-7856	3
TCGA-HT-7857	2
TCGA-HT-7858	2
TCGA-HT-7860	2
TCGA-HT-7873	5
TCGA-HT-7874	3
TCGA-HT-7875	2
TCGA-HT-7877	2
TCGA-HT-7879	5
TCGA-HT-7880	2
TCGA-HT-7881	3
TCGA-HT-7882	5
TCGA-HT-7884	3
TCGA-HT-7902	3
TCGA-HT-8010	2
TCGA-HT-8011	4
TCGA-HT-8012	2
TCGA-HT-8013	4
TCGA-HT-8015	
TCGA-HT-8018	4
TCGA-HT-8019	
TCGA-HT-8104	3
TCGA-HT-8105	4
TCGA-HT-8106	4
TCGA-HT-8107	3
TCGA-HT-8108	3
TCGA-HT-8109	2
TCGA-HT-8110	4
TCGA-HT-8111	3
TCGA-HT-8113	5
TCGA-HT-8114	3

TCGA-HT-8558	2
TCGA-HT-8563	4
TCGA-HT-8564	3
TCGA-HT-A4Ds	
TCGA-HT-A4Dv	2
TCGA-HT-A5R5	3
TCGA-HT-A5R7	3
TCGA-HT-A5Ra	3
TCGA-HT-A5Rb	2
TCGA-HT-A5RC	3
TCGA-HT-A61A	3
TCGA-HT-A61B	2
TCGA-HT-A61C	3
TCGA-HT-A614	4
TCGA-HT-A615	3
TCGA-HT-A616	2
TCGA-HT-A617	2
TCGA-HT-A618	4
TCGA-HT-A619	3
TCGA-DU-8158	4
TCGA-DU-8162	2
TCGA-DU-8163	2
TCGA-DU-8164	3
TCGA-DU-8165	4
TCGA-DU-8166	5
TCGA-DU-8167	3
TCGA-DU-8168	3
TCGA-DU-A5TP	4
TCGA-DU-A5TR	5
TCGA-DU-A5TS	4
TCGA-DU-A5TT	3
TCGA-DU-A5TU	4
TCGA-DU-A5TW	3
TCGA-DU-A5TY	6
TCGA-EZ-7264A	4
TCGA-FG-5962	4
TCGA-FG-5963	
TCGA-FG-5964	4
TCGA-FG-5965	
TCGA-FG-6688	3
TCGA-FG-6689	3
TCGA-FG-6690	3
TCGA-FG-6691	3
TCGA-FG-6692	3
TCGA-FG-7634	4
TCGA-FG-7637	3
TCGA-FG-7641	2
TCGA-FG-7643	4
TCGA-FG-8186	5
TCGA-FG-8189	3
TCGA-FG-A4Mt	2

TCGA-FG-A4Mu	4
TCGA-FG-A60k	3
TCGA-Ht-7467	7
TCGA-Ht-7468	4
TCGA-Ht-7469	
TCGA-Ht-7470	1
TCGA-Ht-7471	3
TCGA-Ht-7472	2
TCGA-Ht-7473	2
TCGA-Ht-7474	2
TCGA-Ht-7475	3
TCGA-Ht-7476	2
TCGA-Ht-7477	3
TCGA-Ht-7478	6
TCGA-Ht-7479	2
TCGA-Ht-7480	2
TCGA-Ht-7481	3
TCGA-Ht-7482	2
TCGA-Ht-7483	2
TCGA-Ht-7485	2
TCGA-Ht-7601	3
TCGA-Ht-7602	3
TCGA-Ht-7603	2
TCGA-Ht-7604	2
TCGA-Ht-7605	2
TCGA-Ht-7606	3
TCGA-Ht-7607	
TCGA-Ht-7608	5
TCGA-Ht-7609	3
TCGA-Ht-7610	2
TCGA-Ht-7611	3
TCGA-Ht-7616	4
TCGA-Ht-7620	3
TCGA-Ht-7676	2
TCGA-Ht-7677	3
TCGA-Ht-7680	2
