# Supplementary material for: Identifying the Hub Genes of Glioma Peritumoral Brain Edema Using Bioinformatical Methods
Source: Brain Sci. 2022 Jun 19;12(6):805. doi: 10.3390/brainsci12060805 (PMC9221376; doi:10.3390/brainsci12060805)
Supplement: Supplementary file 1 [file brainsci-12-00805-s001.zip › Supplementary file S2.pdf]

VASARI MR Feature KEY
(corresponds to Rev 9 of webpage 10/30/08)

Feature number	Name	Description	Options
**F1**	**Tumor Location**	Location of lesion geographic epicenter (not all areas of involvement)	0 = -<br>1 = Frontal<br>2 =Temporal<br>3=Insular<br>4=Parietal<br>5=Occipital<br>6=Brainstem<br>7=Cerebellum
**F2**	**Side of Tumor Epicenter**	Side of lesion epicenter	0= -<br>1=Right<br>2=Center/Bilateral<br>3=Left
**F3**	**Eloquent Brain**	Does the geographic center or the enhancing component involve eloquent cortex (motor, language, vision) or key underlying white matter?	0= -<br>1=None<br>2=Speech motor<br>3=Speech receptive<br>4=Motor<br>5=Vision
**F4**	**Enhancement Quality**:	[None, Mild, Moderate, Marked] Qualitative degree of contrast enhancement is defined as having all or portions of the tumor that demonstrate significantly higher signal on the postcontrast T1W images compared to precontrast T1W images	0= -<br>1=None<br>2=Mild/Minimal<br>3=Marked/Avid
**F5**	**Proportion Enhancing**:	[indeterminate, none (0%), <5%, 6-33%, 34-67%, 68-95%, >95%, All (100%)]. What	0= -<br>1= n/a<br>2=None (0%)<br>3= <5%<br>4= 6-33%

		proportion of the entire tumor is enhancing. (Assuming that the entire abnormality may be comprised of: (1) an enhancing component, (2) a non-enhancing component, (3) a necrotic component and (4) a edema component.)	5= 34-67%<br>6= 68-95%<br>7= >95%<br>8=All (100%)<br>9= Indeterminate
**F6**	**Proportion nCET**	[indeterminate, none (0%), <5%, 6-33%, 34-67%, 68-95%, >95%, All (100%)]. What proportion of the entire tumor is non-enhancing? Nonenhancing tumor is defined as regions of T2W hyperintensity (less than the intensity of cerebrospinal fluid, with corresponding T1W hypointensity) that are associated with mass effect and architectural distortion, including blurring of the gray-white interface.(Assuming that the the entire abnormality may be comprised of: (1) an enhancing component, (2) a non-enhancing component, (3) a necrotic	0= -<br>1= n/a<br>2=None (0%)<br>3= <5%<br>4= 6-33%<br>5= 34-67%<br>6= 68-95%<br>7= >95%<br>8=All (100%)<br>9= Indeterminate

		component and (4) a edema component.)	
**F7**	**Proportion Necrosis**	[indeterminate, none (0%), <5%, 6-33% , 34-67%, 68-95%, >95%, All (100%)]. (Necrosis is defined as a region within the tumor that does not enhance or shows markedly diminished enhancement, is high on T2W and proton density images, is low on T1W images, and has an irregular border). (Assuming that the the entire abnormality may be comprised of: (1) an enhancing component, (2) a non-enhancing component, (3) a necrotic component and (4) a edema component.)	0= -<br>1= n/a<br>2=None (0%)<br>3= <5%<br>4= 6-33%<br>5= 34-67%<br>6= 68-95%<br>7= >95%<br>8=All (100%)<br>9= Indeterminate
**F8**	**Cyst(s)**	Cysts are well defined, rounded, often eccentric regions of very bright T2W signal and low T1W signal essentially matching CSF signal intensity, with very thin, regular, smooth, nonenhancing or regularly enhancing walls, possibly with thin, regular, internal	0= -<br>1= No<br>2= Yes

		septations.</	
**F9**	**Multifocal or Multicentric**	Multifocal is defined as having at least one region of tumor, either enhancing or nonenhancing, which is not contiguous with the dominant lesion and is outside the region of signal abnormality (edema) surrounding the dominant mass. This can be defined as those resulting from dissemination or growth by an established route, spread via commissural or other pathways, or via CSF channels or local metastases, whereas Multicentric are widely separated lesions in different lobes or different hemispheres that cannot be attributed to one of the previously mentioned pathways. Gliomatosis refers to generalized neoplastic transformation of the white matter of most of a hemisphere.	0 = -<br>1= n/a<br>2= Multifocal<br>3= Multicentric<br>4= Gliomatosis
**F10**	**T1/FLAIR RATIO**	Tumor feature summary. [Mixed,	0= -<br>1= Expansive

		expansive or infiltrative]. Expansive = size of pre-contrast T1 abnormality (exclusive of signal intensity) approximates size of FLAIR abnormality. Mixed = Size of T1 abnormality **moderately** less than FLAIR envelope; Infiltrative = Size of pre-contrast T1 abnormality **much smaller** than size of FLAIR abnormality. (Use T2 if FLAIR is not provided)	(T1~FLAIR)<br>2= Mixed (T1<FLAIR)<br>3= Infiltrative (T1<<FLAIR)
**F11**	**Thickness of enhancing margin**	The scoring is not applicable if there is no contrast enhancement. If most of the enhancing rim Is thin, regular, and has homogenous enhancement the grade is thin. If most of the rim demonstrates nodular and/or thick enhancement, the grade is thick. If there is only solid enhancement and no rim, the grade is None.	0= -<br>1= n/a<br>2= None<br>3= Thin<br>4= Thick/solid
**F12**	**Definition of the enhancing margin**	The scoring is not applicable (NA) if there is no contrast enhancement. Assess if most of the outside margin	0= -<br>1= n/a<br>2= Well-defined<br>3= Poorly-defined

		of the enhancement is well defined or poorly defined.	
F13	**Definition of the non-enhancing margin (e.g. Grade III)**	If most of the outside nonenhancing margin of the tumor is well defined and smooth (geographic), versus if the margin is ill-defined and irregular	0= -<br>1= n/a<br>2= Smooth<br>3= Irregular
F14	**Proportion of Edema**	[indeterminate, none (0%), <5%, 6-33%, 34-67%, 68-95%, >95%, All (100%)]. What proportion of the entire abnormality is vasogenic edema? (Edema should be greater in signal than than nCET and somewhat lower in signal than CSF. Pseudopods are characteristic of edema). (Assuming that the the entire abnormality may be comprised of: (1) an enhancing component, (2) a non-enhancing component, (3) a necrotic component and (4) a edema component.)	0= -<br>1= n/a<br>2=None (0%)<br>3= <5%<br>4= 6-33%<br>5= 34-67%<br>6= 68-95%<br>7= >95%<br>8=All (100%)<br>9= Indeterminate
F15	**Edema Crosses Midline**	Edema spans white matter commissures extending into contralateral hemisphere. (exclusive of herniated ipsilateral	0= -<br>1= n/a<br>2= No<br>3= Yes

		tissue)	
**F16**	**Hemorrhage:**	Intrinsic hemorrhage in the tumor matrix. Any intrinsic foci of low signal on T2WI or high signal on T1WI. (Use Bo image if necessary for confirmation.)	0= -<br>1= No<br>2= Yes
**F17**	**Diffusion:**	Predominantly facilitated or restricted diffusion in the enhancing or nCET portion of the tumor. (Based on ADC map). Equivocal is neither. No ADC, use no-images. Proportion of tissue not relevant.	0= -<br>1= No image<br>2= Facilitated<br>3= Restricted<br>4=Neither/equivocal
**F18**	**Pial invasion:**	Enhancement of the overlying pia in continuity with enhancing or non-enhancing tumor	0= -<br>1= No<br>2= Yes
**F19**	**Ependymal invasion:**	Invasion of any adjacent ependymal surface in continuity with enhancing or non-enhancing tumor matrix	0= -<br>1= No<br>2= Yes
**F20**	**Cortical involvement**	Non-enhancing or enhancing tumor extending to the cortical mantle, or cortex is no longer distinguishable relative to subjacent tumor.	0= -<br>1= No<br>2= Yes
**F21**	**Deep WM invasion**	Enhancing or nCET tumor extending into the internal capsule or brainstem.	0= -<br>1= No<br>2= Yes

**F22**	**nCET tumor Crosses Midline:**	nCET crosses into contralateral hemisphere through white matter commissures (exclusive of herniated ipsilateral tissue).	0= -<br>1= n/a (no nCET)<br>2= No<br>3= Yes
**F23**	**Enhancing tumor Crosses Midline:**	Enhancing tissue crosses into contralateral hemisphere through white matter commisures (exclusive of herniated ipsilateral tissue).	0= -<br>1= n/a<br>2= No<br>3= Yes
**F24**	**Satellites:**	A satellite lesion is an area of enhancement within the region of signal abnormality surrounding the dominant lesion but not contiguous in any part with the major tumor mass.	0= -<br>1= No<br>2= Yes
**F25**	**Calvarial remodeling:**	Erosion of inner table of skull (possibly a secondary sign of slow growth)	0= -<br>1= No<br>2= Yes
**F26**	**Extent of resection of enhancing tumor:**	[indeterminate, none (0%), <5%, 6-33% , 34-67%, 68-95%, >95%, All (100%)]. Using the first postoperative scan (contrast-enhanced MR imaging) assessed for tumor residual. Estimate the proportion of enhancing tumor removed. Total	0= -<br>1= n/a<br>2=None (0%)<br>3= <5%<br>4= 6-33%<br>5= 34-67%<br>6= 68-95%<br>7= >95%<br>8=All (100%)<br>9= Indeterminate

		resection of component should be scored 100%. Subtotal resection of enhancing tissue should be scored accordingly.	
**F27**	**Extent resection of nCET**	[indeterminate, none (0%), <5%, 6-33%, 34-67%, 68-95%, >95%, All (100%)]. Using the first postoperative scan (contrast-enhanced MR imaging) assessed for tumor residual. Estimate the proportion of non-enhancing tumor removed. Total resection of component should be scored 100%. Subtotal resection of enhancing tissue should be scored accordingly.	0= -<br>1= n/a<br>2=None (0%)<br>3= <5%<br>4= 6-33%<br>5= 34-67%<br>6= 68-95%<br>7= >95%<br>8=All (100%)<br>9= Indeterminate
**F28**	**Extent resection of vasogenic edema:**	[indeterminate, none (0%), <5%, 6-33%, 34-67%, 68-95%, >95%, All (100%)]. Using the first postoperative scan (contrast-enhanced MR imaging) assessed for tumor residual. Estimate the proportion of edema removed. Total resection of enhancing nidus should be scored 100%. Subtotal resection of enhancing tissue should be scored	0= -<br>1= n/a<br>2=None (0%)<br>3= <5%<br>4= 6-33%<br>5= 34-67%<br>6= 68-95%<br>7= >95%<br>8=All (100%)<br>9= Indeterminate

		accordingly.	
**F29 & F30**	**Lesion Size**	Largest perpendicular (x-y) cross-sectional diameter of T2 signal abnormality (longest dimension X perpendicular dimension) measured on a single axial image only.	0= -<br>1= <0.5cm<br>2= 0.5 cm<br>3= 1.0 cm<br>4= 1.5 cm<br>5= 2.0 cm<br>6= 2.5 cm<br>7= 3.0 cm<br>8= 3.5 cm<br>9= 4.0 cm<br>10= 4.5 cm<br>11= 5.0 cm<br>12 = 5.5 cm<br>13= 6.0 cm<br>14= 6.5 cm<br>15= 7.0 cm<br>16= 7.5 cm<br>17= 8.0 cm<br>18= >8.0cm
